# Deep learning-based automated segmentation of resection cavities on postsurgical epilepsy MRI

**DOI:** 10.1016/j.nicl.2022.103154

**Published:** 2022-08-17

**Authors:** T. Campbell Arnold, Ramya Muthukrishnan, Akash R. Pattnaik, Nishant Sinha, Adam Gibson, Hannah Gonzalez, Sandhitsu R. Das, Brian Litt, Dario J. Englot, Victoria L. Morgan, Kathryn A. Davis, Joel M. Stein

**Affiliations:** a Department of Bioengineering, School of Engineering & Applied Science, University of Pennsylvania, Philadelphia, PA 19104, USA; b Center for Neuroengineering and Therapeutics, University of Pennsylvania, Philadelphia, PA 19104, USA; c Department of Computer Science, University of Pennsylvania, Philadelphia, PA 19104, USA; d Department of Neurology, Perelman School of Medicine, University of Pennsylvania, Philadelphia, PA 19104, USA; e Department of Radiology, Perelman School of Medicine, University of Pennsylvania, Philadelphia, PA 19104, USA; f Department of Neurological Surgery, Vanderbilt University Medical Center, Nashville, TN 37232, USA; g Department of Radiology and Radiological Sciences, Vanderbilt University Medical Center, Nashville, TN 37232, USA; h Department of Biomedical Engineering, Vanderbilt University Medical Center, Nashville, TN 37232, USA; i Institute of Imaging Science, Vanderbilt University Medical Center, Nashville, TN 37232, USA

**Keywords:** Postoperative MRI, Temporal lobe epilepsy, Resection cavity, Automated segmentation, Convolutional neural network, Hippocampal remnant

## Abstract

Accurate segmentation of surgical resection sites is critical for clinical assessments and neuroimaging research applications, including resection extent determination, predictive modeling of surgery outcome, and masking image processing near resection sites. In this study, an automated resection cavity segmentation algorithm is developed for analyzing postoperative MRI of epilepsy patients and deployed in an easy-to-use graphical user interface (GUI) that estimates remnant brain volumes, including postsurgical hippocampal remnant tissue. This retrospective study included postoperative T1-weighted MRI from 62 temporal lobe epilepsy (TLE) patients who underwent resective surgery. The resection site was manually segmented and reviewed by a neuroradiologist (JMS). A majority vote ensemble algorithm was used to segment surgical resections, using 3 U-Net convolutional neural networks trained on axial, coronal, and sagittal slices, respectively. The algorithm was trained using 5-fold cross validation, with data partitioned into training (N = 27) testing (N = 9), and validation (N = 9) sets, and evaluated on a separate held-out test set (N = 17). Algorithm performance was assessed using Dice-Sørensen coefficient (DSC), Hausdorff distance, and volume estimates. Additionally, we deploy a fully-automated, GUI-based pipeline that compares resection segmentations with preoperative imaging and reports estimates of resected brain structures. The cross-validation and held-out test median DSCs were 0.84 ± 0.08 and 0.74 ± 0.22 (median ± interquartile range) respectively, which approach inter-rater reliability between radiologists (0.84–0.86) as reported in the literature. Median 95 % Hausdorff distances were 3.6 mm and 4.0 mm respectively, indicating high segmentation boundary confidence. Automated and manual resection volume estimates were highly correlated for both cross-validation (r = 0.94, p < 0.0001) and held-out test subjects (r = 0.87, p < 0.0001). Automated and manual segmentations overlapped in all 62 subjects, indicating a low false negative rate. In control subjects (N = 40), the classifier segmented no voxels (N = 33), <50 voxels (N = 5), or a small volumes<0.5 cm^3^ (N = 2), indicating a low false positive rate that can be controlled via thresholding. There was strong agreement between postoperative hippocampal remnant volumes determined using automated and manual resection segmentations (r = 0.90, p < 0.0001, mean absolute error = 6.3 %), indicating that automated resection segmentations can permit quantification of postoperative brain volumes after epilepsy surgery. Applications include quantification of postoperative remnant brain volumes, correction of deformable registration, and localization of removed brain regions for network modeling.

## Highlights

• We developed an open-source algorithm for resection cavity segmentation.
• Algorithm performance (0.76–0.84 DSC) approached interrater reliability (0.84 DSC).
• This tool can be used to estimate postoperative remnant volumes (e.g. hippocampus).
• All code and model weights are available: https://github.com/penn-cnt/DeepResection.

## 1 Introduction

Epilepsy is a neurological disorder characterized by recurrent seizures, affecting sixty-five million people worldwide (Kwan and Brodie, 2000). Temporal lobe epilepsy (TLE) is the most common form of epilepsy, with a prevalence of 8.9 cases per 100,000 people per year in the US (Asadi-Pooya et al., 2017). Surgical removal of the epileptic focus is the recommended treatment for drug-resistant TLE, however only about 60 % of patients experience seizure-freedom one year postoperatively (Wiebe et al., 2001, Lee et al., 2005, Mohammed et al., 2012, de Tisi et al., 2011, Cohen-Gadol et al., 2006). A variety of approaches have been used to better predict surgical outcome, including quantitative assessments of resection extent (Bonilha and Keller, 2015, Galovic et al., 2019, Taylor et al., 2018) and modelling the surgical procedure on preoperative functional or structural networks (Taylor et al., 2018, Sinha et al., 2017). However, many of these methods rely on manual segmentation of the resection zone and automated methods for quantifying resection extent would be of significant interest to the epilepsy clinical and neuroimaging research communities.

Retrospective studies attempting to predict surgical outcome of TLE patients use a variety of manual methods which are susceptible to bias due to inter-rater variability. Simple measures, like the inclusion of particular brain structures (e.g. hippocampus, piriform cortex) in the resection zone, have been identified as positive predictors of postoperative seizure freedom (Galovic et al., 2019, Noulhiane et al., 2006). More complex methods mimic surgical resection on brain network models to predict postoperative seizure freedom (Jirsa et al., 2017, Khambhati et al., 2016). The resected brain regions are often determined through manual segmentation or visual inspection (Sinha et al., 2017, Kini et al., 2019). Time-consuming and error-prone manual methods for determining resected tissue limit clinical adoption of these tools. An automated method for delineating resection cavities would be of substantial clinical and research interest, with potential to increase the accuracy of predictive models, evaluate alternative surgical strategies, and improve patient outcomes.

Recent advances in convolutional neural networks (CNNs) have led to vast improvements in classification and segmentation of medical imaging (Yao et al., 2020). Neural network architectures designed for segmentation, such as U-Nets, have been successfully applied to problems from a wide range of specialties, including radiology, pathology, and dermatology (Ronneberger et al., 2015). Additionally, new deep-learning enabled neuroimaging software packages have dramatically reduced processing time for tasks such as brain parcellation (Tustison et al., 2021). A primary goal for many of these tools is the automation of tedious, time-consuming tasks in medicine (Chassagnon et al., 2020). In epilepsy patient care, predictive models of resection extent have not been adopted clinically in part due to their reliance on manual segmentation of resections or visual inspection by researchers, which is time-consuming and variable across individuals and institutions. The lack of automated resection segmentation methods prevents quantitative neuroimaging analyses from being integrated into epilepsy patient care (Duncan et al., 2016).

Therefore, the goal of our study was to develop a fully automated method for segmenting resection volumes and quantifying resected brain tissues, particularly hippocampal remnant volumes, in TLE patients. This tool can evaluate successful removal of surgical targets and has potential to improve predictive models of surgical outcome. Additionally, we present a graphical user interface (GUI) that allows users who are not familiar with machine learning to easily apply the model to their data. We demonstrate that the model can segment the resection zone and estimate which brain regions were removed in under 5 min, permitting easy integration into a clinical workflow. We openly share all code, including the machine learning model, GUI, and statistical analyses to facilitate clinical translation of our work.

## 2 Materials and methods

### 2.1 Data collection

For model training and cross-validation, T1-weighted images (N = 45) were collected from temporal lobe epilepsy (TLE) patients that underwent surgery at the Hospital of the University of Pennsylvania (HUP) or Vanderbilt University Medical Center (VUMC). Internal Review Boards of each institution approved this study, and all patients gave informed consent. Patients at HUP (N = 22) were imaged primarily using a Siemens 3 T scanner with the following T1-weighted sequence parameters: 1 mm isotropic, TE = 3.87 ms, TR = 1.62 s, and flip-angle = 15. Patients at VUMC (N = 23) were imaged using a Phillips 3 T scanner and T1-weighted sequence parameters were 1 mm isotropic, TE = 4.61 ms, TR = 8.9 ms, and flip-angle = 8. All images were collected at least 5 months postoperatively to avoid *peri*-surgical swelling. Inclusion criteria were: 1) TLE patients who underwent resection or ablation, 2) whole-brain, isotropic T1-weighted imaging at least 5 months postoperatively, and 3) only one contiguous resection site. Preoperative T1-weighted imaging using the same scanner and sequence parameters were available for 36 of the 45 patients.

After initial model development, we aggregated additional data to evaluate model performance on a held-out test set and tune the algorithm for use in extratemporal patients. For the held-out test set, we collected 17 T1-weighted images from TLE patients. To tune the model to extratemporal patients, we collected 16 T1-weighted images from patients with resections outside the temporal lobe. To increase sample size, our inclusion criteria for these two sets were relaxed to allow for anisotropic imaging, partial fields of view, and patients with multiple surgical sites. Additionally, we collected a control dataset (N = 40) consisting of T1-weighted images from participants at HUP (N = 20) and VUMC (N = 20). Each institution’s control set contained 10 preoperative images from TLE patients (i.e. patients without a resection) and 10 images from healthy participants.

### 2.2 Data preprocessing

The resection site in each postoperative T1-weighted image was manually segmented in ITK-SNAP (Yushkevich et al., 2006) and reviewed by a board certified neuroradiologist with 8 years of experience (JMS). Each 3D volumetric image was normalized to a standard intensity range [0–1], and 2D slices in each view (axial, coronal, and sagittal) were output as 256x256 Portable Networks Graphic (PNG) files for training.

### 2.3 Model architecture

A majority vote ensemble algorithm using three models trained separately on axial, coronal, and sagittal slices, respectively, was used to segment resections. In the majority vote ensemble, a voxel would be included in the segmentation if it was labeled by at least 2 of the 3 classifiers (i.e. 2 + votes). The same U-Net CNN architecture was used to train each model (Ronneberger et al., 2015). Model construction and training was carried out using the Keras API with TensorFlow backend (Abadi et al., 2016). The model training script was adapted from an open-source U-net segmentation project (Erickson and Cai, 2020) to our model architecture and run on an independent server using a Titan-X GPU. The U-Net architecture consists of an encoder that captures contextual information and a decoder that captures localization information to output a predicted mask. Our model architecture replaced the traditional U-Net encoder with the EfficientNet B1 network encoder backbone, and initial encoder weights were pre-trained on ImageNet data (Deng et al., 2010, Tan and EfficientNet, 2019).

### 2.4 Model training

Each model performed binary segmentation of resections (i.e. 1 = resected, 0 = not resected) on axial, coronal, or sagittal slices of T1-weighted images. During model training, 5-fold cross-validation was employed with data divided into training, validation, and test sets (3:1:1 split). All 2D slices for a given subject were contained within a single set (i.e. training, validation, or testing). The 5-fold approach permits each subject in the dataset to be included in the held-out test set once. Models were trained for 50 epochs using the Adam optimizer, a learning rate of 1e-4, and a batch size of 16. Data augmentation was employed during training to increase model generalizability (Perez and Wang, 2017). Augmentation included random horizontal and vertical flips, rotations up to 10 degrees, and horizontal and vertical shifts up to 10 % of image width and height.

### 2.5 Post processing

Segmentations output by the CNN underwent three post processing steps: 1) assembly of 3D volumes from 2D slice segmentations, 2) majority vote to combine axial, sagittal, and coronal, segmentations, and 3) connected components analysis to remove isolated voxels.

### 2.6 Model evaluation

**Fig. 1:**
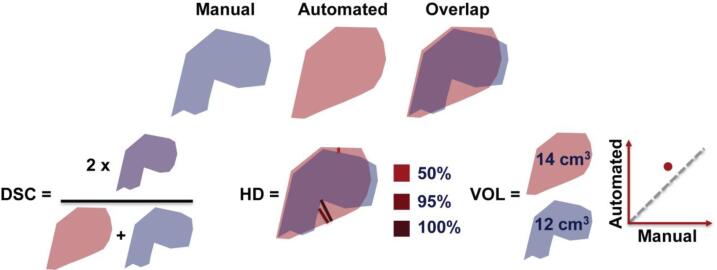
**Schematic of evaluation metrics of classifier performance.** Three metrics were applied: Dice-Sørensen coefficient (DSC), Hausdorff distance (HD), and manual versus automated segmented volumes (VOL). DSC quantifies the overlap between manual and resected segmentations in a range of 0 to 1. HD quantifies the farthest distance between the boundary points. The number of voxels and voxel size quantifies the volume. The classifier performance was optimized by maximizing the DSC.

Performance was evaluated across all cross-validation folds as well as the held-out test dataset. Segmentation performance was primarily evaluated using the Dice-Sørensen coefficient (DSC) (Dice, 1945). DSC measures the overlap between manual segmentation *X* and automated segmentation *Y* (Fig. 1), by computing: $\mathrm{DSC}=\frac{2\left|X\cap Y\right|}{\left|X\right|+\left|Y\right|}$. Performance for DSC ranges from 0 (no overlap) to 1 (perfect match). Hyperparameter optimization was driven by DSC maximization in the validation set. To assess model generalizability to novel images, all reported DSCs were calculated on held-out test datasets.

Subtle changes to performance measures can result in significant differences when ranking algorithms (Maier-Hein et al., 2018). To provide a holistic view of model performance, we report multiple metrics and descriptive statistics. We report two secondary measures, Hausdorff distance and total resection volume (Fig. 1). Hausdorff distance compares actual and predicted segmentation boundaries and reports the distances between adjacent boundary points. This measure characterizes the segmentation border reliability. Several variants of the Hausdorff distance are reported in the literature (Bakas et al., 2018, Gau et al., 2020); we report the 95 % Hausdorff distance, which is more robust to outliers. Additionally, the relationship between manual and automated resection volumes was plotted for each subject and we report the Pearson’s correlation coefficient and mean absolute error (MAE) between these variables.

### 2.7 Model tuning to extratemporal cases

In initial model development, we constrained our target population to only patients with temporal lobe resections. While the temporal lobe is the most common surgical site, epilepsy patients can also have frontal, parietal, and occipital lobe resections. In an exploratory analysis, we tuned our model to perform extratemporal segmentations using a limited dataset of 16 cases (frontal: N = 10, parietal: N = 7, temporal: N = 3, occipital: N = 1, Note: some cases have multiple resections). Patients were separated into training (N = 10) and testing (N = 6) sets. The temporal lobe segmentation model weights were unfrozen and retrained for 50 epochs using the Adam optimizer, a learning rate of 1e-4, and a batch size of 16. All reported metrics were assessed on the held-out test set.

### 2.8 Quantifying surgical remnants

**Fig. 2:**
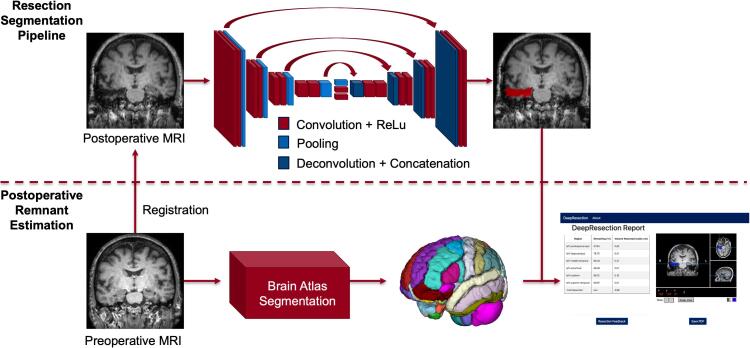
**Pipelines for automated resection segmentation and quantification of postsurgical volume estimates**. The resection segmentation pipeline uses a U-Net architecture (top) and produces a 3D binary mask of resected tissue. To quantify postoperative remnant volumes (bottom), the preoperative image was segmented into brain regions. The intersection of the resection and anatomical brain segmentations were used to generate a resection report.

Postoperative remnant volumes, such as the hippocampal remnant, have predictive value for TLE surgical outcome (Noulhiane et al., 2006). We further developed a pipeline that estimates postoperative remnant brain structures. The pipeline takes a patient’s preoperative and postoperative T1 brain MRI as input and generates a PDF report or an interactive web-based report of estimated resection impact on brain structures (Fig. 2). Preoperative imaging was coregistered to post-operative imaging and segmented into brain regions using the Desikan–Killiany–Tourville (DKT) atlas with subcortical parcellations using the deep-learning enabled toolkit, Advanced Normalization Tools Python (ANTsPyNet) (Tustison et al., 2021, Avants et al., 2009, Klein and Tourville, 2012;6:171.). Images were coregistered using a symmetric normalization transformation, with cross-correlation as the optimization metric and cost-function masking of the resection zone to mitigate image distortion (Brett et al., 2001). Proper image coregistration was verified manually and any subjects with significant distortion (N = 1) were excluded. The resection cavity was segmented both manually and using the automated algorithm described here for comparison. The intersection of preoperative brain segmentations and the postoperative resection segmentation was used to estimate remnant brain volumes. We correlated hippocampal remnant volume estimates between manual and automated resection segmentation methods. The postsurgical hippocampal remnant tissue was not manually segmented, but rather estimated using the manual resection segmentation.

### 2.9 Code availability

All code related to model design and postoperative volume estimation can be found at: https://github.com/penn-cnt/DeepResection. Code related to statistical analysis can be found at: https://github.com/penn-cnt/DeepResection_Statistical_Analysis.

## 3 Results

### 3.1 Patient characteristics

Our main dataset included 45 patients who underwent surgery for localization-related epilepsy across two institutions, the Hospital of the University of Pennsylvania (HUP, N = 22) and Vanderbilt University Medical Center (VUMC, N = 23). Patients were age-matched across institutions (HUP: 39.2 ± 12.0 years, VUMC: 39.5 ± 12.7 years) and were treated with either anterior temporal lobectomy (ATL, N = 28), selective amygdalohippocampectomy (SAH, N = 15), or hippocampal laser interstitial thermal therapy (LITT, N = 2). There was no significant difference between institutions for the patients’ gender, age of seizure onset, age at surgery, side of seizure surgery, age at scan, or disease duration. There was a significant difference in the surgical approach between institutions (*X^2^* = 21.99, p < 0.0001, chi-square test), as patients were only treated with SAH at one center. Demographic information is provided in Table 1.

**Table 1:** Patient demographic information. Abbreviations: Hospital of the University of Pennsylvania (HUP), Vanderbilt University Medical Center (VUMC), anterior temporal lobectomy (ATL), selective amygdalohippocampectomy (SAH), Laser interstitial thermal therapy (LITT), standard deviation (SD).

	VUMC	HUP	Total
Sex (female / male)	12 / 11	16 / 6	28 / 17
Surgical approach (ATL / SAH / LITT)	7 / 15 / 1	21 / 0 / 1	28 / 15 / 2
Side of surgery (left / right)	6 / 17	11 / 11	17 / 28
Age at surgery (years, mean ± SD)	37.5 ± 11.5	36.6 ± 12.8	37.1 ± 12.1
Age at scan (years, mean ± SD)	39.2 ± 12.0	39.5 ± 12.7	39.3 ± 12.2
Age at onset (years, mean ± SD)	19.5 ± 10.6	18.9 ± 12.2	19.1 ± 11.6
Duration (years, mean ± SD)	15. 6 ± 8.7	21.8 ± 15.7	19.9 ± 14.1

### 3.2 Primary performance measure (DSC)

**Fig. 3:**
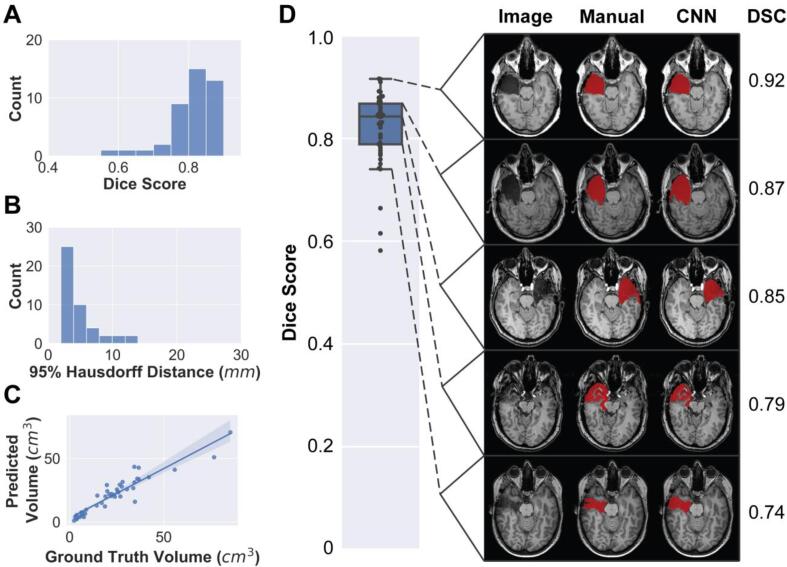
**Classifier accuracy across the cross-validation cohort.** Here we report model performance on the held-out test sets (N = 45) during cross-validation. (A) Dice-Sørensen coefficient (DSC), 0.84 ± 0.08 (median ± interquartile range). (B) 95 % Hausdorff distance, 3.61 ± 2.64 mm (median ± interquartile range). (C) Pearson correlation between predicted and manually segmented volumes (r = 0.94, p < 0.0001). (D) Representative manual and automated segmentations from each quartile of the Dice score distribution. Segmentations are overlaid on the T1-weighted images, with their associated DSC on the right-hand side.

The majority vote model was trained using 5-fold cross-validation and accuracy is reported using the per-scan DSC for held out subjects in the cross-validation test sets (Table 2). The average test DSC across all scans was 0.82 ± 0.07 (mean ± standard deviation), with a median DSC of 0.84 and interquartile range of 0.08 (Fig. 3A). The maximum DSC achieved by the classifier for a given patient was 0.92, while the minimum score was 0.58. To illustrate the range of segmentation quality, Fig. 3D shows examples of manual segmentations and corresponding predicted labels at each quartile of the DSC distribution. The majority vote classifier modestly outperformed individual axial, sagittal, and coronal classifiers as well as more stringent or relaxed voting schemes (Figure S1).

**Table 2:** Cross-validation (CV) and held-out test set results. Two metrics are reported, Dice-Sørensen coefficient (DSC) and 95% Hausdorff distance (HD).

Fold	DSC		95 % Hausdorff Distance (mm)	
	Median / Interquartile Range	Mean ± Standard Deviation	Median / Interquartile Range	Mean ± Standard Deviation
1	0.85 / 0.15	0.81 ± 0.10	4.00 / 6.23	5.78 ± 3.66
2	0.83 / 0.10	0.82 ± 0.05	2.96 / 2.20	3.79 ± 1.45
3	0.85 / 0.03	0.83 ± 0.10	3.55 / 1.60	3.64 ± 1.44
4	0.83 / 0.06	0.82 ± 0.04	4.00 / 1.94	4.73 ± 2.20
5	0.83 / 0.04	0.84 ± 0.03	3.66 / 4.00	5.72 ± 3.95
**All CV**	**0.84 / 0.08**	**0.82 ± 0.07**	**3.61 / 2.64**	**4.73 ± 2.90**
**Held-out Test set**	**0.74 / 0.22**	**0.68 ± 0.18**	**4.14 / 11.81**	**9.78 ± 9.13**

### 3.3 Secondary performance measures (Hausdorff distance & volume)

Two secondary performance measures were assessed, Hausdorff distance and predicted resection volume. Hausdorff distance quantifies the maximal distance between analogous boundary points in ground truth and predicted segmentations. In our analyses we have included the 95 % Hausdorff distance, which has been utilized as an accuracy measure in prominent segmentation challenges (Deng et al., 2010). In our dataset the median 95 % Hausdorff distance was 3.61 ± 2.64 mm (Fig. 3B). This indicates that 95 % of boundary points were within 3.61 mm of the target.

When comparing manual and automated segmentation volumes, predicted volumes were slightly smaller (21.0 ± 14.7 ml) than manually labeled segmentations (22.9 ± 17.6 ml), with a significant difference detected in a pairwise *t*-test (p = 0.044, t = -2.07). There was a strong correlation (Fig. 3C) between the manual and automated volumes (r = 0.94, p < 0.0001, MAE = 4.2 cm^3^).

### 3.4 False negatives & false positives

The classifier’s false negative and false positive rates of lesion detection were also assessed. A false negative was defined as the algorithm having no segmentation overlap with the manual segmentation. A false positive was defined as inappropriate segmentation in a control subject. For all resection patients, the classifier correctly lateralized their resection to the appropriate hemisphere and the predicted resection overlapped the ground truth label. This indicates a low false negative rate for lesion detection (effectively 0 %). To evaluate the potential for false positives, we applied the classifier to 40 control subjects (20healthycontrolsand20preoperativeepilepsypatientswithnoresection). In 33 control subjects, no resection segmentation was produced. In 5 subjects, <50 voxels were segmented, while the remaining 2 subjects had a small volume (<0.5 cm^3^ or 500 voxels) of hypointense temporal lobe tissue segmented (Figure S2). Given the small segmentation sizes, these false positives can be effectively screened out by applying a segmentation size threshold. Importantly, the low false positive rate indicates the classifier is sensitive to the presence of a resection, not simply localizing the temporal lobe and producing an average resection mask as output.

### 3.5 Lesion size relationships

Previous studies have found a relationship between lesion size and classifier accuracy as measured by DSC and percent volume difference (PVD) between predicted and manual segmentations (Gau et al., 2020). To understand whether lesion size contributed to classifier accuracy or PVD error, we partitioned subjects into small (N = 17) and large (N = 28) resection groups using the same threshold (﻿﻿17.92 ml) previously reported (Tan and EfficientNet, 2019). In our model, we found the average DSC was greater for large resections (large = 0.84, small = 0.79, p = 0.03, t = 2.24, two-sample *t*-test) and that PVD was higher for small resections (large = 16.3, small = 25.1, p = 0.049, t = 2.02, two-sample *t*-test), both of which indicate a larger error for smaller resection segmentations. Predicted and actual volumes for small resections were not significantly different (mean volume: actual = 7.2 ml, predicted = 6.9 ml, p = 0.63, t = 0.49, two-sample *t*-test), however large resections tended towards under-segmentation (mean volume: actual = 32.5 ml, predicted = 29.5 ml, p = 0.050, t = 2.05, two-sample *t*-test).

### 3.6 Held-out test set performance

**Fig. 4:**
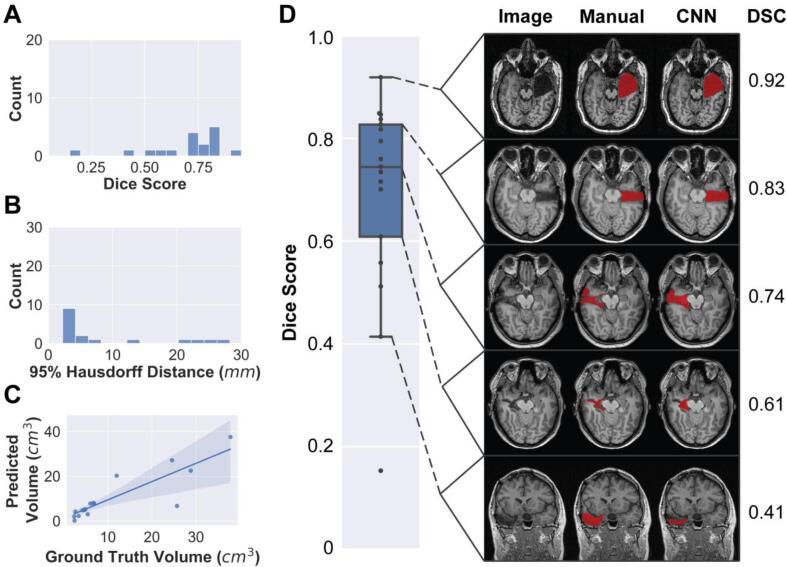
**Classifier accuracy across the held-out cohort.** Here we report model performance on the final held-out test set (N = 17) collected after model development. (A) Dice-Sørensen coefficient (DSC), 0.74 ± 0.22 (median ± interquartile range). (B) 95 % Hausdorff distance, 4.04 ± 10.32 mm (median ± interquartile range). (C) Pearson correlation between predicted and manually segmented volumes (r = 0.87, p < 0.0001). (D) Representative manual and automated segmentations from each quartile of the Dice score distribution. Segmentations are overlaid on the T1-weighted images, with their associated DSC on the right-hand side.

In addition to the held-out test sets used in the cross-validation approach, we compiled a separate held-out set containing 17 TLE patients after model development. We evaluated model performance on this test set using the same metrics used in the cross-validation set. Model performance decreased slightly in the held-out test set (Table 2). The median DSC was 0.74 with an interquartile range of 0.22 (Fig. 4A), compared to 0.84 ± 0.06 in the cross-validation set. The median 95 % Hausdorff distance increased from 3.61 ± 2.64 mm (median ± interquartile range) to 4.04 ± 10.32 mm (Fig. 4B). The comparison of predicted and manually segmented volumes remained similar between the two sets (cross-validation: r = 0.94, p < 0.0001, held-out test set: r = 0.87, p < 0.0001). Examples comparing the manual and automated segmentations from throughout the DSC distribution are seen in Fig. 4D. Although model performance decreased slightly in the held-out test set, it is important to note that inclusion criteria were relaxed for the held-out test set to increase the sample size, which may have impacted our results.

### 3.7 Comparing surgical approaches

Next, we compared the algorithm’s performance between patients treated with SAH (N = 14) and ATL (N = 29) to determine if one surgical approach accounted for a greater degree of model error. Patients treated with LITT were excluded from statistical analysis, as only two patients were in this group. There was no significant difference in our primary performance measure, DSC (ATL: 0.83 ± 0.07, SAH: 0.81 ± 0.06, mean ± standard deviation, p = 0.25, t = 1.15). Patients treated with LITT had slightly lower DSC (0.61 and 0.74), possibly caused by the low number of training samples and hyperintense coagulative necrosis in ablation cavities (LaRiviere and Gross, 2016). There was no difference in the 95 % Hausdorff distance between ATL and SAH groups (ATL: 4.5 ± 2.0 mm, SAH: 4.2 ± 2.9 mm, mean ± standard deviation, p = 0.71, t = 0.37). As expected, both the ground truth volumes (ATL: 29.2 ± 13.7 cm^3^, SAH: 7.2 ± 3.9 cm^3^, mean ± standard deviation, p < 0.001, t = 5.66) and predicted volumes (ATL: 27.6 ± 11.1 cm^3^, SAH: 6.7 ± 3.4 cm^3^, mean ± standard deviation, p < 0.001, t = 6.63) were significantly larger for patients treated with ATL.

### 3.8 Visual inspection

**Fig. 5:**
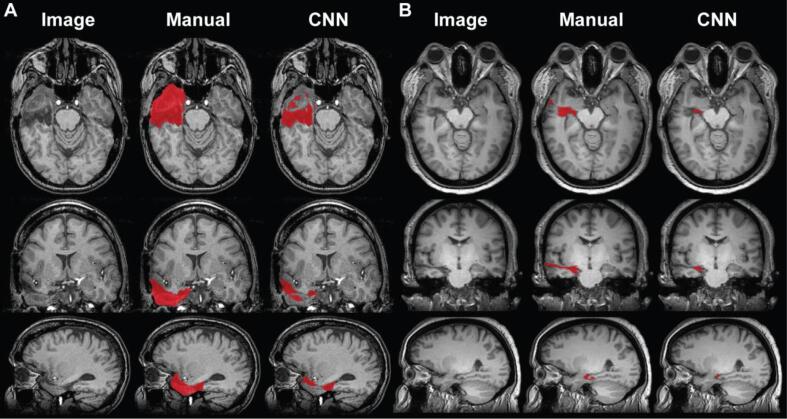
**Example output from the lowest scoring segmentations.** (A) The lowest segmentation overlap case in the cross-validation set was a subject with hyperintense blood product in the resection cavity. (B) In the held-out test set, the lowest segmentation overlap was a SAH case, where surgical tracts were manually segmented but not included in the automated segmentation.

The largest sources of segmentation error were small resection volumes, hyperintense material in the resection cavity, surgical tracts, boundaries between resections and ventricles, and resections that extended into parietal regions. The relationship between resection size and segmentation accuracy is detailed in section *3.5 Lesion size relationships*. Hyperintense material in the resection cavity (e.g. blood products, LITT coagulative necrosis (LaRiviere and Gross, 2016), and residual tissue fragments) were included in manual resections but sometimes ignored by the classifier (Fig. 5A). There were two examples of patients treated with LITT (DSC: 0.61 and 0.74), and the hyperintense material was not segmented in both cases. In patients treated with SAH, the surgical tracts were included in manual segmentations but ignored in some automated segmentations (Fig. 5B). Additional sources of error included atypical resections that extended posteriorly and difficulty delineating the boundary of resections adjacent to the lateral ventricles (Figure S3).

### 3.9 Model tuning to extratemporal cases

The model tuned using extratemporal cases was able to segment the resection site in 5 of 6 test set patients, with one frontal lobe resection missed (Figure S4D). Resection segmentation was at least partially successful in the remaining subjects, with a median DSC of 0.75 ± 0.23 (Figure S4A). In one subject with non-contiguous frontal and temporal lobe resections, only the frontal lobe resection was segmented (Figure S4D). The median 95 % Hausdorff distance was 10.35 ± 32.59 mm (median ± interquartile range) (Figure S4B). A similar relationship between predicted and actual volumes was seen in this limited dataset (r = 0.80, p = 0.054, Figure S4C). Example of all test set segmentations can be seen in Figure S4D. We do not recommend using the model weights from this exploratory analysis but offer this as preliminary evidence that the classifier can be tuned to work with extratemporal resections given sufficient data.

### 3.10 Quantifying surgical remnants

**Fig. 6:**
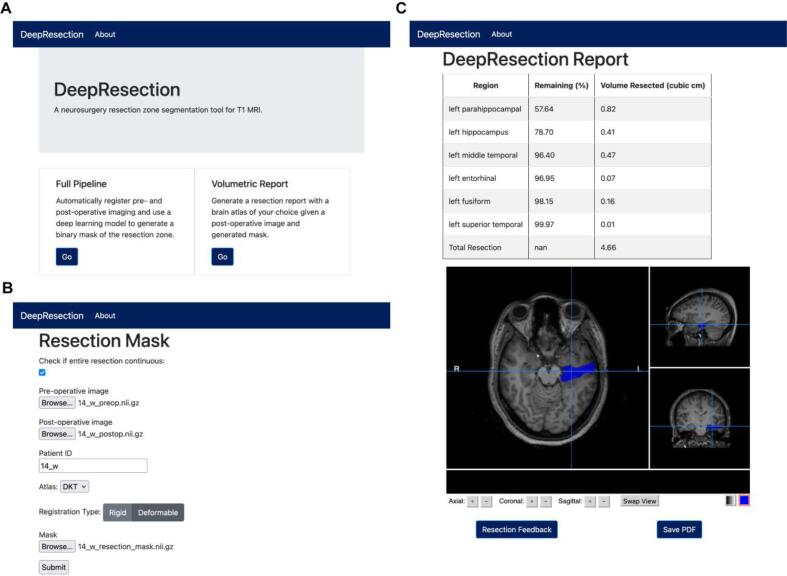
**Graphical User Interface (GUI) for estimating surgical remnants.** Here we illustrate the GUI interface developed for estimating resection remnants on a selective amygdalohippocampectomy patient. (A) In the first panel, the user selects to run the full pipeline or run the analysis using a resection volume they generated. (B) The user then uploads the required images and selects their desired registration and segmentation parameters. (C) The pipeline outputs a table of affected regions by percentage resected and provides an interactive visualization of the resection segmentation for manual review and quality control.

The web application that deploys our pipeline consists of a set of sequential web pages where users can upload pre- and post-operative MR images, visualize the automated segmentation, and save a report estimating resected brain regions to their local desktop. The landing screen lets the user choose between applying the full pipeline to their data using our automated segmentation algorithm or uploading a manually generated segmentation and visualizing the report. The full pipeline consists of pre- and post-operative image registration (Tustison et al., 2021), pre-operative segmentation using the DKT brain atlas with subcortical structures (Klein and Tourville, 2012;6:171.), and resection segmentation using our described model. The report page consists of a table listing affected brain regions, a 3D resection mask viewer, and optional user feedback (Fig. 6). The report table provides the total resection volume and lists affected brain regions by percentage resected. An embedded 3D mask viewer allows the user to make a quality assessment of the predicted mask against the post-operative image. To assess the feasibility of deploying the full pipeline to users, we computed the time elapsed for running the web application. The average run time was 4 min and 19 s, and all run times were<5 min. A detailed user manual for running the web application can be found in the GitHub repository.

**Fig. 7:**
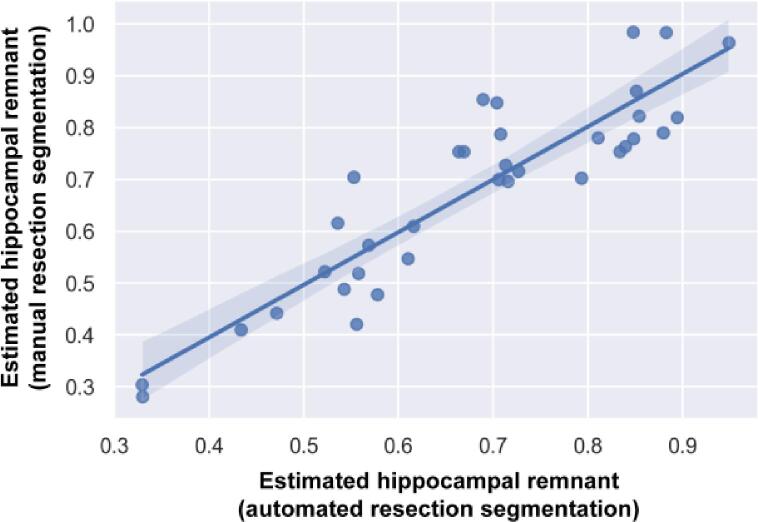
**Strong correlation between remnant estimates using automated and manual methods.** We compared hippocampal remnant estimates using automated and manual resection segmentations. Automated and manual estimates are significantly correlated (r = 0.90, p < 0.0001) and have a mean absolute error of 6.3 %.

Hippocampal remnants have previously been associated with worse surgical outcomes (Noulhiane et al., 2006). We compared hippocampal remnant volumes using the manual and predicted resection segmentations for a subset of 36 patients with available preoperative imaging. One subject was excluded due to poor image registration. Fig. 7 illustrates the correlation between hippocampal remnant estimates made using the manual labels and automated segmentation (r = 0.90, p < 0.0001, mean absolute error = 6.3 %).

## 4 Discussion

We present a deep-learning method to fully automate resection cavity segmentation in postoperative temporal lobe epilepsy patients. Fully automated segmentation provides significant time advantages over manual and semi-automated methods (Ronneberger et al., 2015, Yushkevich et al., 2006, Atsina et al., 2016). Our method has several key advantages. First, we trained our model explicitly on TLE patients, who are most frequently operated on for drug-resistant epilepsy. Second, our resection labels are based on gold standard clinical practice (manually segmented by a neuroradiologist with 8 years of experience and subspecialization in epilepsy imaging). Third, we included multi-site data from two epilepsy centers, thus demonstrating potential for multi-center studies. Fourth, by employing an ensemble of 2D CNNs trained on different views of the brain, our segmentation algorithm utilizes the whole 3D volume without using an explicitly 3D model. 3D CNNs learn exponentially more parameters than 2D CNNs, so compared to 3D CNNs, our algorithm avoids high computational training costs and can learn better on modestly sized training datasets. Fifth, we provide a fast method for volumetric analysis of resected brain regions for post-hoc analysis. Sixth, we incorporate a graphical user interface (GUI) for easy interpretation of segmentation quality. We demonstrate the clinical utility of our algorithm by quantifying postoperative remnant structures, which have been shown to predict long-term surgical outcome.

One advantage of the present study is epilepsy patient data was used during model training. A previous study applied lesion_GNB, a stroke segmentation classifier, to the resection cavity segmentation problem in epilepsy patients (Gau et al., 2020, Griffis et al., 2016). While lesion_GNB demonstrated some utility in segmenting resections (median DSC 0.58), our classifier achieved greater segmentation accuracy. The discrepancy in classifier performance may be caused in part by differences in features between the pathologies, such as lesion intensity and surrounding edema. Resection cavity segmentation has also been attempted in glioblastoma multiforme (GBM) patients (Ermiş et al., 2020). Here the classifier was trained explicitly on GBM patient data and classifier performance (median DSC 0.83) was similar to trained radiation oncologists (median DSC 0.85). These studies highlight the importance of developing disease specific classifiers or applying transfer learning to fine-tune models for specific pathologies (Pan and Yang, 2010).

Other approaches to boost classifier performance include the incorporation of simulated training data. Pérez-García et al. recently reported the development of EPISURG, a self-supervised resection segmentation classifier that uses exclusively simulated resection data (Brett et al., 2001). Their classifier achieved a median DSC of 0.805 using 2074 simulated resections, which surpassed their classifier trained using 133 manual labels (median DSC 0.653). This illustrates the significant performance gains possible through innovative data augmentation. However, it is important to note that all versions of their model report false negatives, meaning in some subjects the resection was entirely missed. This is likely due in part to broad inclusion criteria, but false negatives may also be occurring because important features such as gliosis, blood products and brain shift are not included in simulated data.

Researchers have also explored automated methods for resection zone segmentation that do not rely on machine learning. Casseb et al. developed ResectVol (Casseb et al., 2021), an SPM-based program that relies on differences in tissue probability maps between preoperative and postoperative images to identify the resection site and estimate removed brain structures. The authors achieved promising results, with a median DSC of 0.77 and significant correlation between automated and manual resection volumes (r = 0.8, p < 0.001). However, conventional image analysis approaches require longer processing times and may be less resilient to contrast changes associated with ancillary pathology, such as gliosis and edema, or different surgical approaches, such as LITT.

Automating quantification of resected tissue can catalyze progress in at least four fields of epilepsy and neuroimaging research. First, automated resection segmentation can be used to predict brain network reorganization after surgery. Many patients who are initially seizure free after surgery have a seizure relapse in the long term, possibly due to changes in the networks over time (de Tisi et al., 2011). Quantifying brain structures remaining after surgery, by reliably delineating tissues resected, is crucial in predicting such changes and determining patients who are likely to have late recurrences (de Tisi et al., 2011, Morgan et al., 2020). Second, modeling proposed surgeries improves prediction of surgical outcomes (Galovic et al., 2019, Sinha et al., 2021). Precise delineation of resected tissue would facilitate retrospectively analyzing factors associated with seizure outcomes after surgery. Third, our clinical application protocols can be applied prospectively to quantify network changes for alternate surgical strategies before carrying out an actual surgery (Taylor et al., 2018, Kini et al., 2019). This is particularly useful for patients who are likely to have poor surgical outcomes or in patients in which the site of resection is close to the eloquent cortex. Finally, surgical resection is known to cause brain shift as cerebral spinal fluid is lost and the resection cavity collapses, leading to positional changes between pre- and post-operative timepoints (Narasimhan et al., 2020). As such, deformable registration would be required to properly align these images for comparison. However, surgical resection also produces significant errors during deformable registration, resulting in erroneous extrusion of nearby tissue into the resection cavity (Brett et al., 2001). Our algorithm can be integrated into neuroimaging pipelines to automatically perform cost-function masking of the resection zone, allowing for more accurate processing of postoperative images.

Our study had several limitations, including a use of strict inclusion criteria, poor representation of laser ablations and extratemporal resections, and a single image rater. Our initial approach was to use strict inclusion criteria because a relatively homogenous patient population would likely maximize classifier performance. This limits classifier generalizability to TLE patients and restricts available training data to a smaller sample size. Our dataset size is fairly modest for training deep learning models. However, in future studies, criteria will be relaxed to include patients with resections outside the temporal lobe, different surgical approaches, and a broader range of clinical imaging sequences. Furthermore, laser ablations and extratemporal resection patients were poorly represented in our dataset, which resulted in lower segmentation accuracy for these patients. We have provided a preliminary analysis demonstrating the ability to tune our model to segment extratemporal resections, though a larger sample size will be necessary to produce a robust model. Increasing representation of laser ablations and extratemporal resections, either as actual or simulated data, could improve classification for these patients. An additional study limitation was that manual segmentations were only available from a single neuroradiologist. Having a single rater prevents the assessment of inter-rater reliability (IRR) in our study; however IRR has been assessed for resection segmentation by other groups and results are fairly consistent across studies (median DSC 0.84–0.86) (Ermiş et al., 2020, Pérez-García et al., 2020).

## 5 Conclusion

In conclusion, we developed a fully automated method for segmenting the resection cavity and quantifying brain regions removed in TLE surgical patients. Our method performance approaches IRR between radiologists while significantly reducing manual input and can be deployed in an easy-to-use GUI. Automated resection cavity segmentation methods have important implications for predictive models of surgical interventions and consistency across multi-center trials. We openly share all code and model weights for our classifier to enable acceleration towards clinical translation and improvement of epilepsy patient care.

## 6 Disclosures

Brian Litt is an unpaid Scientific Advisor to 4Catalyzer. Dr. Litt is a Co-Founder of Liminal Science, which was recently acquired by HealthCor Catalio, and Dr. Litt has ownership in the resulting company. Joel M. Stein has received support from sponsored research agreements with Hyperfine Research, Inc. and consulting income from Centaur Diagnostics, Inc. Both Dr. Stein's and Dr. Litt's interactions with these companies, and those of their trainees, are performed in strict accordance with the policies and conflict of interest management rules of the University of Pennsylvania and are reviewed annually.

## CRediT authorship contribution statement

**T. Campbell Arnold:** Conceptualization, Data curation, Methodology, Software, Formal analysis, Writing – original draft, Writing – review & editing. **Ramya Muthukrishnan:** Data curation, Methodology, Software, Formal analysis, Writing – original draft, Writing – review & editing. **Akash R. Pattnaik:** Methodology, Formal analysis, Writing – review & editing. **Nishant Sinha:** Methodology, Writing – review & editing. **Adam Gibson:** Data curation, Project administration. **Hannah Gonzalez:** Methodology, Formal analysis. **Sandhitsu R. Das:** Methodology, Writing – review & editing. **Brian Litt:** Writing – review & editing, Supervision, Funding acquisition. **Dario J. Englot:** Data curation, Project administration, Writing – review & editing, Supervision, Funding acquisition. **Victoria L. Morgan:** Data curation, Project administration, Writing – review & editing, Supervision, Funding acquisition. **Kathryn A. Davis:** Data curation, Project administration, Writing – review & editing, Supervision, Funding acquisition. **Joel M. Stein:** Methodology, Writing – review & editing, Supervision.

## Declaration of Competing Interest

The authors declare that they have no known competing financial interests or personal relationships that could have appeared to influence the work reported in this paper.

## Data Availability

Data will be made available on request.
