# Supplementary material for: Deep learning-based automated segmentation of resection cavities on postsurgical epilepsy MRI
Source: Neuroimage Clin. 2022 Aug 17;36:103154. doi: 10.1016/j.nicl.2022.103154 (PMC9402390; doi:10.1016/j.nicl.2022.103154)
Supplement: Supplementary data 1 [file mmc1.docx]

**Supplemental Figures**


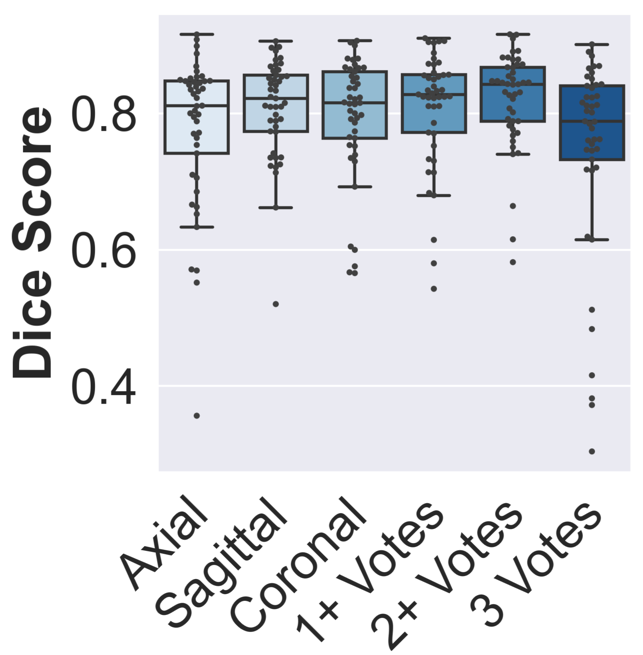


**Figure S1. Comparison of Dice scores across individual and ensemble classifiers.** We applied individual and ensemble voting classifiers to all cross-validation test set subjects. Performance was similar across all classifiers (median 079-0.84 ± 0.08-0.11 interquartile range). The majority vote approach (i.e. 2+ Votes) had the highest Dice score and smallest interquartile range (0.84 ± 0.08).


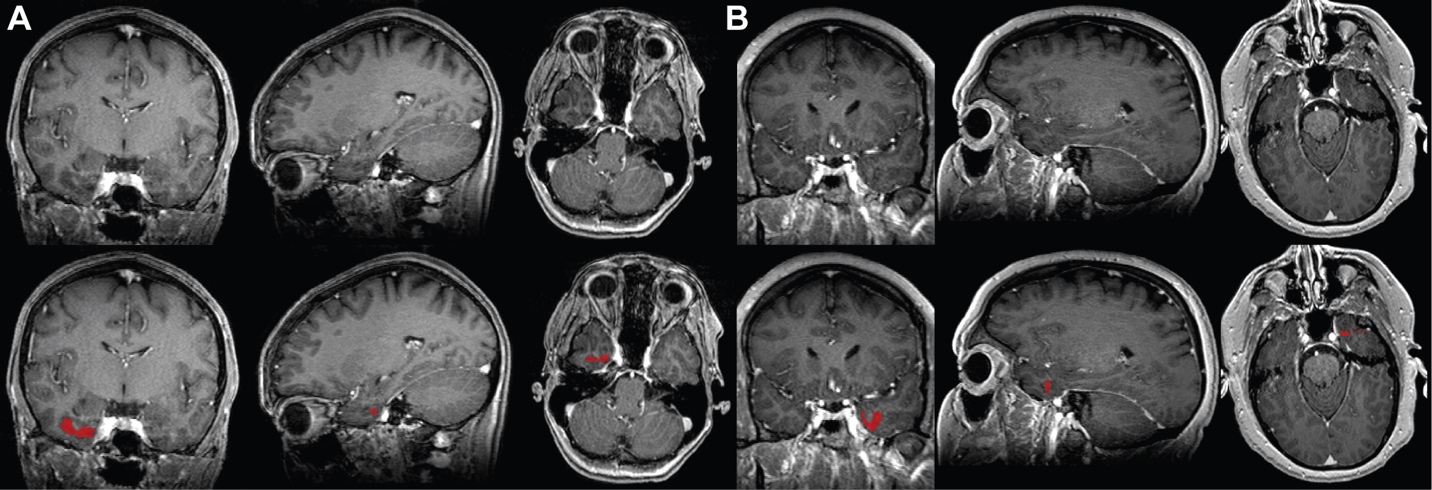


**Figure S2. Two cases of false positive segmentation in preoperative patients.** We applied our segmentation algorithm to 40 participants without resections (20 preoperative TLE patients, 20 healthy controls). The classifier appropriately output no segmentation in 33 cases, segmented less than 50 voxels in 5 cases, and segmented a small volume of A) 0.36 cm^3^ and B) 0.44 cm^3^ in the above cases. A minimum cluster size threshold could be applied to mitigate false positive detections.


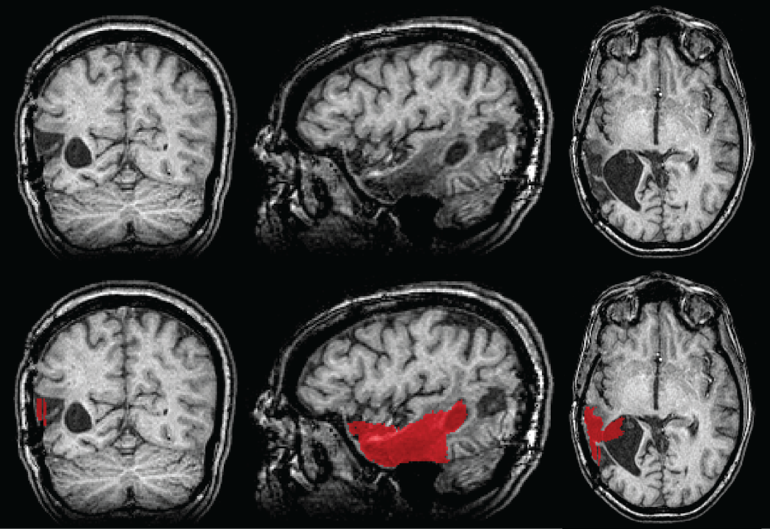


**Figure S3. Undersegmentation of posterior resection regions and ventricular oversegmentation.** In the main dataset, two sources of error were resections that extended posteriorly beyond the typical resection extend and resections that bordered the ventricles. Both issues are visualized in the above subject.


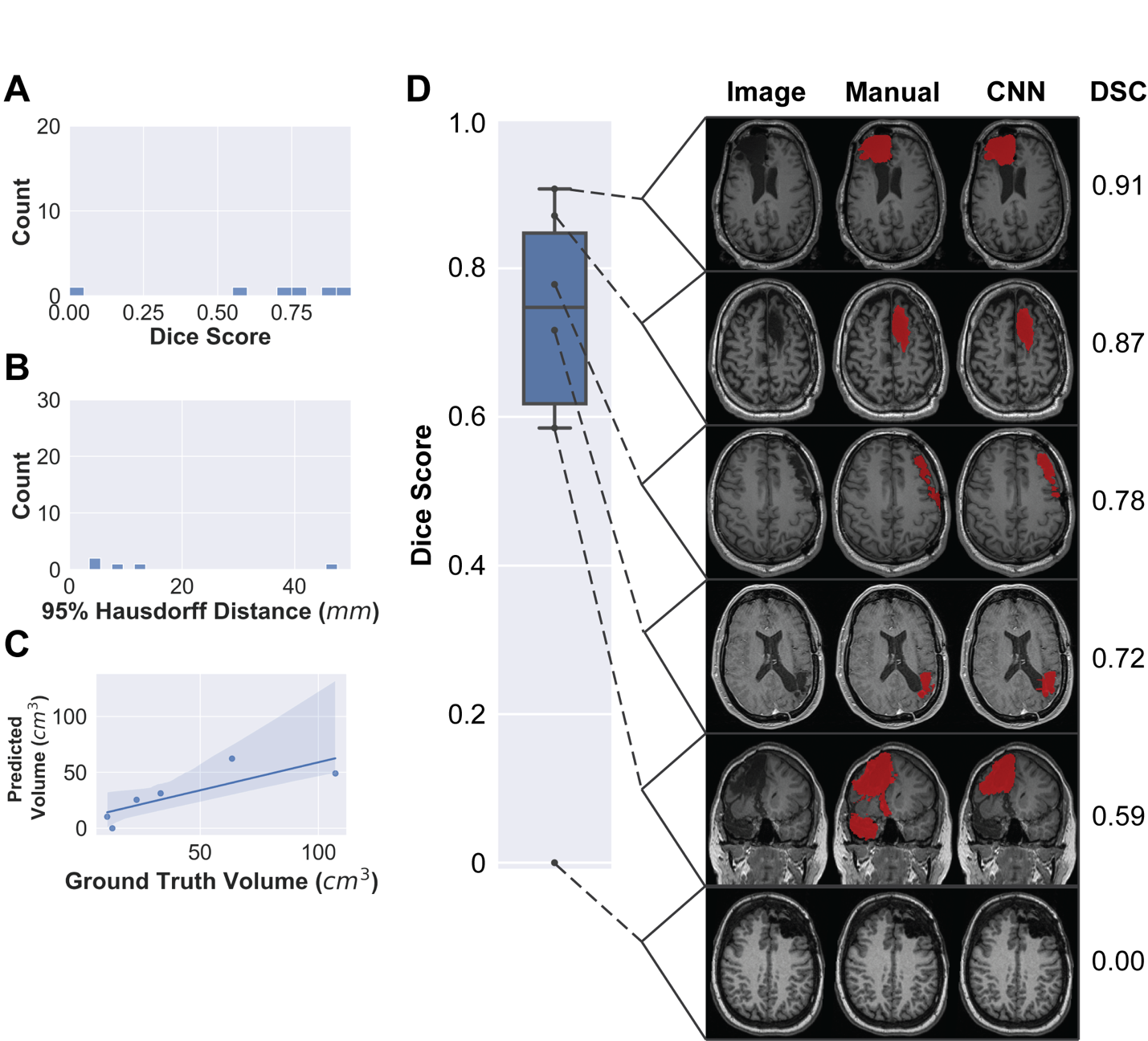


**Figure S4. Tuned classifier accuracy across the extratemporal cohort.** Here we report tuned model performance on a held-out test set (N = 6) of extratemporal resection patients. (A) Dice-Sørensen coefficient (DSC), 0.75 ± 0.23 (median ± interquartile range). (B) 95% Hausdorff distance, 10.35 ± 32.59 mm (median ± interquartile range). (C) Pearson correlation between predicted and manually segmented volumes (r = 0.80, p = 0.054). (D) Examples slices from all manual and automated segmentations from each patient in the extratemporal held-out test set. Segmentations are overlaid on the T1-weighted images, with their associated DSC on the right-hand side.
